# Adenosine triphosphate-based chemotherapy response assay-guided chemotherapy in unresectable colorectal liver metastasis

**DOI:** 10.1038/bjc.2011.469

**Published:** 2011-11-08

**Authors:** H Hur, N K Kim, H G Kim, B S Min, K Y Lee, S J Shin, J H Cheon, S H Choi

**Affiliations:** 1 Department of Surgery, Yonsei University College of Medicine, Seoul, Republic of Korea; 2 Department of Pathology, Yonsei University College of Medicine, Seoul, Republic of Korea; 3 Department of Medical Oncology, Yonsei University College of Medicine, Seoul, Republic of Korea; 4 Department of Gastroenterology, Yonsei University College of Medicine, Seoul, Republic of Korea; 5 ISU ABXIS Co., Ltd., Seoul, Korea

**Keywords:** unresectable colorectal liver metastasis, neoadjuvant chemotherapy, adenosine triphosphate-based chemotherapy response assay, treatment response, liver resection

## Abstract

**Background:**

This study aims to evaluate the effectiveness of adenosine triphosphate-based chemotherapy response assay (ATP-CRA)-guided neoadjuvant chemotherapy for increasing resectability in patients with unresectable colorectal liver metastasis.

**Patients and methods:**

Patients were randomised into two groups: Group A was treated by conventional chemotherapy regimen and Group B was treated by chemotherapy regimen according to the ATP-CRA. Three chemotherapeutic agents (5-fluorouracil, oxaliplatin and irinotecan) were tested by ATP-CRA and more sensitive agents were selected. Either FOLFOX or FOLFIRI was administered. Between Group A and B, treatment response and resectability were compared.

**Results:**

Between November 2008 and October 2010, a total 63 patients were randomised to Group A (*N*=32) or Group B (*N*=31). FOLFOX was more preferred in Group A than in Group B (26 out of 32 (81.3%) *vs* 20 out of 31 (64.5%)). Group B showed better treatment response than Group A (48.4% *vs* 21.9%, *P*=0.027). The resectability of hepatic lesion was higher in Group B (35.5% *vs* 12.5%, *P*=0.032). Mean duration from chemotherapy onset to the time of liver resection was 11 cycles (range 4–12) in Group A and 8 cycles (range 8–16) in Group B.

**Conclusion:**

This study showed that tailored-chemotherapy based on ATP-CRA could improve the treatment response and resectability in initially unresectable colorectal liver metastasis.

Colorectal cancer is the second leading cause of cancer death in western countries and liver metastasis from colorectal cancer is the leading cause of cancer-related morbidity and mortality (Boyle and Ferlay, 2005). Nearly half of the patients will develop liver metastases at some point during the course of disease, with 20% having metastases at the time of diagnosis (Jemal *et al*, 2004). Complete resection of all liver metastasis is the only therapy with potential for cure, and the overall 5-year survival rate of 30–35% can be expected after curative liver resection (Choti *et al*, 2002). However, only 10–25% of all patients with liver metastasis received curative liver resection (Lochan *et al*, 2007). The other patients, who did not undergo radical surgery, had undergone conventional chemotherapy or radiofrequency ablation (RFA), but the outcome was inferior to that of the radical surgery (Abdalla *et al*, 2004). Furthermore, median survival without any treatment is 6–12 months (Wagner *et al*, 1984).

Recently, neoadjuvant chemotherapy for patients with unresectable colorectal liver metastasis was performed to decrease the number and size of metastatic lesion and convert to resectable status. The result of conventional chemotherapeutic regimens containing 5-fluorouracil (5-FU), oxaliplatin and irinotecan (FOLFOX or FOLFIRI) have shown 34–56% of response rate and 12–35% of conversion rate (Bismuth *et al*, 1996; Adam *et al*, 2004; Tournigand *et al*, 2004; Alberts *et al*, 2005; Barone *et al*, 2007; Masi *et al*, 2009). On the other hand, newly introduced biological agents, cetuximab (a chimeric human–mouse monoclonal antibody to the epidermal growth factor receptor) and bevacizumab (a chimeric human–mouse monoclonal antibody to the vascular endothelial growth factor) represent a better response rate of 46–80% and conversion rate of about 30–60% (Adam *et al*, 2007; Saltz *et al*, 2008; Bokemeyer *et al*, 2009; Van Cutsem *et al*, 2009; Folprecht *et al*, 2010; Garufi *et al*, 2010). However, these agents have several limitations of use; the relatively high cost for treatment, the difference of response by the genetic status of patients and major adverse side-effects. To overcome these problems, optimal tailored chemotherapy with conventional chemotherapeutic agents is emerging as an alternative option for increasing the response rate and improving treatment outcomes.

The individualised chemotherapeutic regimen for tailored chemotherapy can be chosen by *in vitro* chemosensitivity assay. An *in vitro* chemosensitivity assay has been developed to predict chemotherapy outcomes and evaluate the inhibition rate of tumour growth by various chemotherapy drugs (Wilbur *et al*, 1992; Kurbacher *et al*, 1998). The adenosine triphosphate (ATP)-based assay is a sensitive cytometric assay that evaluates tumour cell viability by measuring the intracellular ATP levels of drug-exposed cells and untreated controls (Kawamura *et al*, 1997; Sharma *et al*, 2003). This test has several advantages to compare with other tests, higher sensitivity to predict the cell viability, easy to distinguish between cancer cells and normal cells and requires only small number of cells (Maehara *et al*, 1987; Petty *et al*, 1995). Several preclinical and clinical studies reported the feasibility and good treatment outcomes of ATP-chemotherapy response assay (ATP-CRA)-guided chemotherapy in ovarian, breast, stomach and lung cancer (Moon *et al*, 2007; Park *et al*, 2007; Han *et al*, 2008; Kim *et al*, 2008). In colorectal cancer, heterogeneity of chemosensitivity of colorectal adenocarcinoma was determined by ATP-CRA in chemotherapeutic agents, including 5-FU, oxaliplatin, irinotecan and so on (Whitehouse *et al*, 2003). However, there are no data about clinical outcomes of ATP-CRA-guided chemotherapy in colorectal cancer.

According to recent clinical practice guideline update by the ASCO, the use of chemotherapy sensitivity and resistance assays can be recommended in the clinical trial setting. As the *in vitro* analytical strategy has potential importance, participation in clinical trials evaluating these technologies including ATP-based assay remains a priority (Burstein *et al*, 2011).

This study was designed to evaluate the feasibility and usefulness of ATP-CRA-guided chemotherapy as neoadjuvant treatment for increasing tumour response and resectability in patients with unresectable colorectal liver metastasis.

## Patients and methods

### Patients

Between November 2008 and October 2009, 63 colorectal cancer patients with unresectable liver metastasis were enrolled in this prospective study at the Severance Hospital, Yonsei University Health System, Seoul, Korea. Our protocol was approved by the Institutional Review Board of Severance Hospital. Eligibility criteria included a histologically confirmed diagnosis of colorectal adenocarcinoma with unresectable liver metastasis; age >18 years old; Eastern Cooperative Oncology Group performance status ⩽2, adequate bone marrow, renal and liver function, no previous chemotherapy or radiotherapy and no history of other malignancies within 5 years. Patients with simultaneous single extrahepatic metastasis were also enrolled as long as their extrahepatic lesion was resectable. All patients were informed of the investigational nature of the studies and they provided written informed consent before enrollment. Patients were randomised for two subgroups using random numbers: (1) Group A was treated by conventionally selected chemotherapy regimen by a medical oncologist. (2) Group B was treated by chemotherapy regimen according to the ATP-CRA results of primary cancer tissue. At least 20 mg of tumour tissue was obtained by rigid sigmoidoscopy with one piece or colonoscopy with six pieces.

### Assessments

The liver metastases were diagnosed by an abdominal CT or by magnetic resonance imaging (MRI). The unresectability of liver metastatic lesions was established through a multidisciplinary assessment performed by the Colorectal Tumor Board of Severance Hospital, which comprises of colorectal and hepatic surgeons, medical and radiation oncologists as well as diagnostic radiologists. Patients considered to have technically unresectable disease had a too small remnant liver volume with relation to the extent of the resection that was needed for complete resection of all metastases. This was defined by the need of a resection leaving less than 25–30% of liver remnant.

### Chemotherapy

All patients were administrated to same chemotherapy regimen, FOLFOX or FOLFIRI. FOLFOX regimen consisted of oxaliplatin 85 mg m^−2^ (2 h infusion) and then leucovorin 200 mg m^−2^ (2 h infusion), followed by 5-FU 400 mg m^−2^ (bolus injection, intravenously) and 5-FU 2400 mg m^−2^ administrated as a 46-h continuous infusion on day 1. FOLFIRI regimen consisted of irinotecan 180 mg m^−2^ (2 h infusion) and then leucovorin 200 mg m^−2^ (2 h infusion) followed by 5-FU 400 mg m^−2^ (bolus injection, intravenously), and 5-FU 2400 mg m^−2^ administrated as a 46-h continuous infusion on day 1. Treatment was administrated every 2 weeks until hepatic resection, evidence of disease progression, unacceptable toxicity or patient refusal. Chemotherapeutic regimen changed in cases of poor responses after the assessment of treatment response.

### ATP-CRA methodology

Adenosine triphosphate-based chemotherapy response assay was performed as described previously (Moon *et al*, 2007). The histological types of the tumour tissues, qualitative and quantitative analyses of cancer cells were evaluated by pathologists. Specimen underwent washing and mincing followed by enzymatic disaggregation using 1.7 units ml^−1^ Dispase (Sigma, St Louis, MO, USA), 10 *μ*g ml^−1^ Pronase (Sigma) and 15 *μ*g ml^−1^ DNase (Sigma) at 37 °C from 12 to 16 h. Isolated cells were separated from tissue fragments by passing through a cell strainer (BD Falcon, Bedford, MA, USA). Tumour cells were separated from dead cells and red blood cells by ficoll (1.077 g ml^−1^) gradient centrifugation at 400 **g** for 15 min. If sufficient amount of cells were isolated, blood-derived normal cells were removed using anti-CD45 antibody-conjugated magnetic beads (Miltenyi Biotech, Auburn, CA, USA; Iinuma *et al*, 2000). The separated tumour cell preparation was suspended in IMDM (Gibco BRL, Rockville, MD, USA) including 10% FBS. The cells were then diluted to a cell concentration between 5000 and 15 000 viable cells per 100 μl for plating into a 96-well Ultra Low Attachment micro-plate (Costar, Cambridge, MA, USA) with or without anti-cancer drugs and cultured for 48 h in the CO_2_ incubator. Treated drug concentrations (TDCs) were determined by preliminary experiment, which exhibit scattered distribution of cell death from each specimen (Bird *et al*, 1985). Drugs were used in triplicates at 0.2 × , 1 × and 5 × TDC. The TDCs used were as follows: 5-FU 10 *μ*g ml^−1^, oxaliplatin 2.9 *μ*g ml^−1^ and Irinotecan 4.7 *μ*g ml^−1^. To measure the ATP level, ATP in the cell lysate was reacted with luciferin and excessive luciferase (Roche, Mannheim, Germany) using Victor 3 multi-label counter (PerkinElmer, Boston, MA, USA). Excel-based raw data were analysed by Report Maker version 1.1 (ISU ABXIS, Seoul, Korea). Briefly, the cell death rate for each drug was calculated as follows: cell death rate (%)=(1−(mean luminescence in treated group/mean luminescence in untreated controls group)) × 100. We have also calculated the chemosensitivity index as the sum of percentage cell death for all concentrations tested (300−sum of % cell death rate at × 0.2, × 1 and × 5 TDC). 5-Fluorouracil was selected as a based chemotherapeutic agent regardless of its tested sensitivity. Between irinotecan and oxaliplatin, the drug with the higher cell death rate was chosen.

### Tumour response evaluation

The assessment of treatment response had carried out every three or four chemotherapy cycles and was used to determine whether the resection of metastatic hepatic lesion is possible or not by using consistent imaging techniques such as CT and MRI. In case of response, the Colorectal Tumor Board performed all of the assessments with regard to tumour responses and if metastases were resectable according to the Response Evaluation Criteria in Solid Tumors during the final period (Therasse *et al*, 2000). Complete response (CR) was defined as the disappearance of all liver metastases, whereas a partial response (PR) was defined as a reduction of at least 30% of the sum of the longest diameter of liver metastases, with no evidence of new lesions or progression of either intra- or extrahepatic lesions. Progressive disease (PD) was defined as an increase of at least 20% of the sum of the longest diameters of liver metastases from the time the treatment started, or the appearance of either new intra- or extrahepatic lesion(s). Stable disease (SD) was indicated when there was neither a sufficient shrinkage to qualify for PR nor a sufficient increase to qualify for PD.

### Surgery

Surgical resection of metastatic lesions was reconsidered during chemotherapy and was performed when technically feasible and potentially curative. Liver metastases were considered resectable when the simultaneous clearing of all metastases was possible with a remnant liver volume of at least 25–30%, regardless of the size or the number of metastases. Radiofrequency ablation was allowed, solely or in association with surgery, to treat few (three or fewer) and small-sized (⩽3 cm) metastasis without remnant lesion. Resection of primary colorectal tumour was performed simultaneously with liver operation. All surgeries were carried out with curative intent.

### Statistical analysis

This study was prospective randomised study and the sample size was calculated. In order to detect a difference between a liver resectability rate of 10% in the conventional chemotherapy group and 40% in the ATP-CRA group with an *α* of 0.05 and a statistical power of 80%, a total sample size of 70 is needed, allowing for a 10% dropout rate (*χ*^2^-test). In total, 35 patients are required in each group. The primary endpoint was the rate of conversion from unresectable liver metastasis to resectable ones. Tumour response and conversion rate was evaluated and compared between Groups A and B. Categorical variables were analysed using the *χ*^2^-test or Fisher's exact test, and continuous variables were analysed using the two-sided *t*-test. Multivariate analysis was carried out using a logistic regression analysis. Statistical analysis was performed using the SPSS software version 12.0 (SPSS Inc., Chicago, IL, USA). A value of *P*<0.05 was considered significant.

## Results

### Patients and tumour characteristics

**Figure 1:**
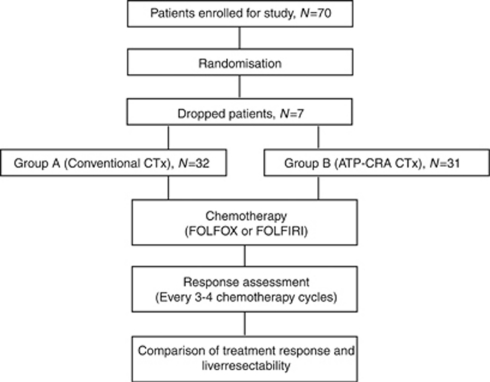
Flow chart demonstrating patients enrollment, treatment and response assessment. ATP-CRA, adenosine triphosphate-chemotherapy response assay; CTx, chemotherapy.

A total of 70 patients were enrolled, however 63 patients could participate in the planned study. Four patients showed chemotherapy toxicity, and chemotherapy was ceased before the first-treatment response evaluation. Three patients dropped out of this study voluntarily in the initial stages (Figure 1). Table 1 shows the characteristics of 63 patients (Group A (*N*=32) and Group B (*N*=31)), including primary tumour and metastasis. The median follow-up period was 12 months in both the groups. The mean age, sex, primary tumour location and mean serum carcinoembryonic antigen showed no significant differences. Extrahepatic metastases were found in four patients in Group A and three patients in Group B, all of which were located in the lung and resectable. The mean number of liver metastatic lesions and the mean sum of tumour size was similar between the two groups.

### Chemotherapy and surgery

FOLFOX was preferred in Group A rather than in Group B (26 out of 32 (81.3%) *vs* 20 out of 31 (64.5%), *P*=0135). Four patients of Group A underwent curative treatment for liver metastasis, including two cases of major hepatectomy, one case of wedge resection and one case of RFA for remnant small lesion. In Group B, 11 patients achieved curative liver resection. Among these patients, five patients underwent major hepatectomy, and segmentectomy or wedge resection was performed in six patients. In three cases of liver resection, RFA was performed together. There was no positive resection margin or remnant tumour in all liver resection cases. The mean duration from chemotherapy onset to the time of liver resection was 11 cycles (range 8–16) in Group A and 8 cycles (range 4–12) in Group B (Table 2). The primary tumour was resected in all patients who underwent curative treatment for liver metastasis.

### Response evaluation and treatment outcomes

Of the 32 patients of Group A, no patient achieved CR of the liver metastases, and 7 patients (21.9%) achieved PR. Thus, the overall response rate was 21.9%. Among these seven patients, four patients (12.5%) underwent liver resection or RFA, one patient is continuing chemotherapy with the same regimen and two patients changed the chemotherapeutic regimen. In total, 15 patients (46.9%) showed SD, and 6 patients are continuing chemotherapy with the same regimen, but 5 patients changed the chemotherapeutic regimen by adding targeting agents and 4 patients changed from FOLFOX to FOLFIRI. Among the 10 patients (31.2%) that showed PD, 8 patients changed their regimen with 7 patients adding targeting agents and 2 patients refused to continue chemotherapy. Four patients (12.5%) underwent curative resection of liver.

Of the 31 patients in Group B, 1 patient achieved CR (3.2%) and 14 patients (45.2%) achieved PR. Thus, the overall response rate was 48.4%. Liver resection was performed for one patient with CR. Among 14 patients with PR, 8 patients underwent liver resection, 3 patients are continuing chemotherapy with the same regimen and 3 patients changed the chemotherapeutic regimen. Eight patients (25.8%) showed SD, and two patients received liver resection, three patients are continuing chemotherapy with the same regimen, but two patients changed the chemotherapeutic regimen by adding targeting agents and one patient changed from FOLFIRI to FOLFOX. Among the eight patients (25.8%) that showed PD, seven patients changed their regimen to either FOLFOX or FOLFIRI and targeting agent was added for one patient. Curative resection of liver metastases could be performed in 11 patients, including 1 patient who had CR. Thus, the resection rate was 35.5%.

**Figure 2:**
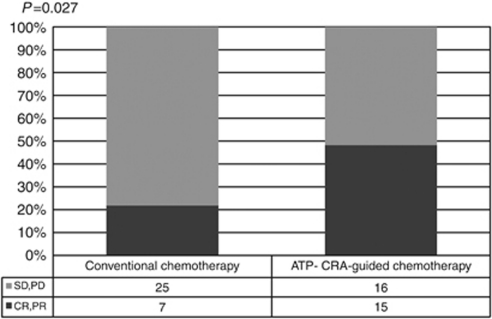
Comparison of treatment response according to chemotherapy methods. ATP-CRA, adenosine triphosphate-chemotherapy response assay; CR, Complete response; PR, Partial response; SD, Stable disease; PD, Progressive disease.

**Figure 3:**
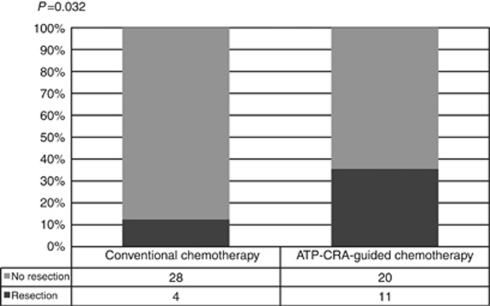
Comparison of liver resectability according to chemotherapy methods. ATP-CRA, adenosine triphosphate-chemotherapy response assay.

Group B showed better treatment response than group A (48.4% *vs* 21.9%, *P*=0.027). The resectability of the hepatic lesion was significantly higher in Group B than in Group A (35.5% *vs* 12.5%, *P*=0.032) (Figures 2, 3).

### Correlation of ATP-CRA results with treatment response and liver resectability

In 31 patients, who received ATP-CRA-based chemotherapy, cell death rates (CDRs) of each drug were analysed. Regarding all patients, mean CDR was 30.5% for 5-FU, 28.2% for oxaliplatin and 26.1% for irinotecan. Mean CDR of 5-FU was significantly higher in patients showing CR or PR than for SD or PD (34.5% *vs* 26.7%, *P*=0.026). Patients who underwent liver resection also demonstrated higher CDR of 5-FU (36.2% *vs* 27.3%, *P*=0.040). Oxaliplatin or irinotecan was selected as a second drug combined with 5-FU by ATP-CRA. When comparing CDR of all second drugs, either oxaliplatin or irinotecan, according to treatment response and liver resectability, CR/PR and liver resection group showed significantly higher CDR (38.1% *vs* 25.7%, *P*=0.001; 39.9% *vs* 27.2%, *P*=0.008). In 20 patients treated with oxaliplatin as second drug, CDR of oxaliplatin was higher in patients showing CR or PR than for SD or PD (41% *vs* 24.5%, *P*=0.013). Liver resection group also demonstrated higher CDR (41.6% *vs* 25.5%, *P*=0.033). Eleven patients who received irinotecan also showed similar results (Table 3).

## Discussion

A number of studies have shown that the unresectable colorectal liver metastasis is not fatal. Indeed, the efficiency of new chemotherapy regimens has increased the number of patients who could be candidates for curative resection of colorectal liver metastasis.

Bismuth *et al* (1996) were the first to evaluate the role of conversion chemotherapy for 330 patients with unresectable colorectal liver metastasis. They observed that an additional 53 (16%) patients could receive a curative surgical resection after FOLFOX. In the following series, 1104 patients with unresectable liver metastasis were treated by 5-FU and leucovorin combined to oxaliplatin, irinotecan or both. Among these 1104 patients, 138 (12.5%) underwent secondary hepatic resection after an average 10 courses of chemotherapy (Adam *et al*, 2004). Recently Adam *et al* (2009) reported the long-term outcome of 184 patients, initially unresectable colorectal liver metastases, who underwent rescue surgery. The 5- and 10-year overall survival rates were 33% and 27%, respectively. Overall, 24 (16%) patients were considered cured; cure defined as disease-free interval ⩾5 years from last hepatic or extrahepatic resection until the last follow-up.

In the series of phase II study, Alberts *et al* (2005) have evaluated 42 patients with unresectable liver-only metastases treated with FOLFOX4. In total, 25 (60%) patients experienced a reduction of tumour and 14 (33%) patients had a complete hepatic resection. Barone *et al* (2007) reported the long-term follow-up data of neoadjuvant chetherapy with FOLFIRI in patients with unresectable colorectal liver metastasis. Of 40 patients, 19 (47.5%) patients experienced an objective response and 13 (32.5%) patients underwent curative liver resection. Median time to progression was 5.2 months for non-resected patients and 52.5 months in resected patients (Barone *et al*, 2007).

In phase III randomised trials, Giacchetti *et al* (2000) have evaluated patients with metastatic colorectal cancer treated with 5-FU and LV with or without oxaliplatin and reported the higher response rate (53% *vs* 16%) and complete resection rate (21% *vs* 17%) in oxaliplatin-added group.

In these series, authors demonstrated that neoadjuvant chemotherapy in patients with initially unresectable liver metastases from colorectal cancer seems a potential curable option. Post-chemotherapy resectability was a significant independent prognostic factor for better survival rate than in patients with no resection. However, it should be underlined that patients presenting disease progression while under chemotherapy had a worse prognosis and the decision to perform liver resection should be considered with caution. In addition, there are some unanswered questions about the optimal neoadjuvant regimen and appropriate cycles before surgery.

The randomised GERCOR study investigated two sequences: FOLFIRI followed by FOLFOX (*N*=109) or FOLFOX followed by FOLFIRI (*N*=111) in inoperable metastatic colorectal cancer (Tournigand *et al*, 2004). In first-line therapy, the response rates were 56% with FOLFIRI *vs* 54% with FOLFOX. Surgical treatment to remove metastasis after first-line therapy was performed in 10 patients (9%) in FOLFIRI arm *vs* 24 patients (22%) in FOLFOX arm. All patients undergoing surgery had liver metastasis except one. The mean number of cycles given before surgery was 12 cycles of FOLFIRI and 10 cycles of FOLFOX. However, curative resection was possible in 8 patients (7%) of FOLFIRI arm *vs* 14 in FOLFOX arm (13%, *P*=0.26). When used as first-line therapy, there was no difference in terms of progression-free survival for FOLFIRI or FOLFOX. There was no difference in terms of overall survival between either arms of the study.

Between FOLFOX and FOLFIRI, there is no conclusive evidence that one particular regimen is superior to the other for increasing resectability in patients with unresectable colorectal liver metastasis. A major systematic review of irinotecan and oxaliplatin in the treatment of advanced colorectal cancer founded that the reported resection rates ranged from 9 to 35% in patients receiving FOLFIRI and from 7 to 51% in patients receiving FOLFOX (Hind *et al*, 2008).

Therefore, the selection of a regimen between FOLFOX and FOLFIRI is an important issue and the evidence and method is needed to find the appropriate regimen for each of the patients. ATP-CRA may be useful to decide the regimen for tailored treatment in neoadjuvant chemotherapy of unresectable colorectal liver metastasis. In our study, the group that was treated by the chemotherapy regimen according to an ATP-CRA result had better response and resectability of metastatic liver lesions than the conventional chemotherapy group. We selected more effective chemotherapeutic agents according to tumour inhibition rate by ATP-CRA. Indeed, patients who achieved good treatment response and underwent liver resection demonstrated the higher CDR in all administered drugs. This may show the potential usefulness of ATP-CRA in individualised chemotherapy.

Standardisation of unresectability criteria will facilitate the evaluation of outcomes of liver resection after neoadjuvant chemotherapy and will provide the optimal treatment for all potential resection candidates. Recently, a consensus group proposed new guidelines for determining unresectability of hepatic metastases. These criteria include: (1) tumour involves more than 70% of liver, or six liver segments; (2) unresectable extrahepatic disease; (3) unfit patients for surgery (Poston *et al*, 2005). In present study, the same criteria are also used to facilitate the interpretation of results. A number of features are identified to be associated with resectability after neoadjuvant treatment. The anatomical distribution of liver metastases and cancer biology was positive prognostic features. The response rate and resectability of conventional chemotherapy group in this study are 21.9% and 12.5%, respectively, and relatively lower than previous studies. These results may be caused by the relatively high number of liver metastases and large tumour size of enrolled patients in this study. Moreover, liver metastases are distributed bilaterally in more than 80% of the patients. Despite the similar characteristics of liver metastases, ATP-CRA-based chemotherapy group showed considerably good results of response rate (48.4%) and resectability (35.5%).

Clinical characteristics including ATP-CRA were analysed to evaluate the association with treatment response and liver resection. The number of liver lesions was smaller in patients who showed a good treatment response and received liver resection. FOLFIRI regimen showed a trend toward a higher good treatment response, but not liver resection. However, all other characteristics except APT-CRA did not show the statistical significance to be associated with treatment response and liver resection (Table 4). Furthermore, according to multivariate logistic regression analysis, ATP-CRA was significantly associated with good treatment response (*P*=0.004, 95% CI: 2.4–96.3) and liver resection (*P*=0.009, 95% CI: 2.0–139.6). The number of liver lesions was another factor for complete or partial treatment response (*P*=0.010, 95% CI: 1.1–1.8), but not significant for liver resection statistically (Table 5). Higher response rates to chemotherapy are associated with higher resectability rates. Thus, optimisation of the preoperative regimens to achieve a high probability of response is critical to the success of surgical resection.

In patients who showed poor response in first-line treatment by chemotherapeutic agent, either FOLFOX or FOLFIRI, the regimen eventually changed to another one or biological agents were added. If they initially received the regimen expected to show good response, they may not miss the opportunity of liver resection without an increase of liver metastasis. In addition, they may reduce the financial burden to use the expensive biological agents. ATP-CRA showed the possibility of tailored treatment regarding chemotherapy in individual patients. Despite the choices of chemotherapy regimen for metastatic colorectal cancer is fewer, either FOLFOX or FOLFIRI, the more sensitive regimen could improve treatment response without disease progression. Good treatment response may lead to curative resection of tumour and better prognosis.

Recent studies have shown the remarkable improvement of tumour response and survival outcomes after first-line treatment with biological agents (cetuximab or bevacizumab) in metastatic colorectal caner (Saltz *et al*, 2008; Bokemeyer *et al*, 2009; Van Cutsem *et al*, 2009). Furthermore, phase II trials that used biological agents in unresectable liver only-disease with endpoints of response rate and secondary resectability were reported. CELIM study of cetuximab plus FOLFOX or FOLFIRI (Folprecht *et al*, 2010) demonstrated high response rates of 68% and 57%, respectively. R0 resection rates of 38% and 30%, respectively, were achieved. In POCHER trial (Garufi *et al*, 2010), cetuximab plus chronomodulated irinotecan, 5-FU, leucovorin, and oxaliplatin also yielded a high response rate of 79% and a secondary resectability rate of 60%. *KRAS* mutational status was shown to be a highly predictive selection criterion in relation to the treatment decision regarding the use of cetuximab.

Although recent results of the biological agents, bevacizumab and cetuximab, have shown that they improve clinical surgical outcomes when added to current first-line regimens in patients with metastatic colorectal cancer, FOLFOX and FOLFIRI are still used in most of the patients with metastatic colorectal cancer as the first-line treatment. Neoadjuvant treatment with bevacizumab may cause major adverse events, such as wound-healing delays, bleeding and defects in liver regeneration at the time of surgery. Hence, the interval between neoadjuvant bevacizumab and hepatic resection is needed. For cetuximab, *KRAS* mutational status should be considered and preferred only in patients with wild-type KRAS tumours. Moreover, because the biological agent alone could not be used as a single agent, the combination therapy with the cytotoxic agent, FOLFOX or FOLFIRI regimen, is needed. Therefore, for more effective chemotherapy to patients, ATP-CRA may be useful to select FOLFOX or FOLFIRI regimen for combination with the biological agents.

Biopsy of the liver metastasis has the risk of cancer cell contamination and dissemination. In most of the cases, primary and metastatic lesion shows the same therapeutic effect by chemotherapy and the same biology. After discussion of this issue, we decided that the primary tissue would be better for being obtained by simple endoscopic biopsy and tested by ATP-CRA.

In conclusion, to provide a potentially curative option for patients with unresectable colorectal liver metastases, there is a clear need for effective neoadjuvant chemotherapy that yields good tumour response leading to secondary resection. Our initial experience suggests a possible approach to improve the treatment response and resectability in patients with unresectable colorectal liver metastasis by tailored-chemotherapy based on ATP-CRA. To obtain the evidence of better survival after this treatment modality, long-term follow-up and more accumulated data are needed. Furthermore, multi-institutional phase III randomised controlled trials are required so that ATP-CRA can be used as a feasible and effective assay for individualised chemotherapy in unresectable colorectal liver metastases.

## Figures and Tables

**Table 1:** Patient and tumour characteristics

**Variables**	**Group A, *N*=32**	**Group B, *N*=31**	***P*-value**
Mean age (year; range)	55 (34–79)	58 (44–75)	0.145
			
*Sex*			
Male	18 (56.3)	17 (54.8)	0.910
Female	14 (43.8)	14 (45.2)	
Mean FU months (range)	12 (10–18)	12 (10–18)	0.966
			
*Primary tumour location*			
Rectum	13 (40.6)	13 (41.9)	0.916
Colon	19 (59.4)	18 (58.1)	
			
*Distant metastasis*			
Liver only	28 (87.5)	28 (90.3)	1.000
Liver and lung	4 (12.5)	3 (9.7)	
			
*Metastatic tumour of liver*			
Mean number (range)	7 (2–30))	9 (2–20)	0.174
Mean size (cm; range)	10.3 (2.5–33)	9.6 (5.0–35)	0.680
Location			
Unilobe	6 (18.8)	5 (16.1)	0.784
Bilobe	26 (81.3)	26 (83.9)	
Mean serum CEA (ng ml^−1^; range)	83.8 (1.0–338)	63.1 (2.7–314)	0.350
			
*Chemotherapy regimen*			
FOLFOX	26 (81.3)	20 (64.5)	0.135
FOLFIRI	6 (18.8)	11 (35.5)	

Abbreviations: CEA=carcinoembryonic antigen; FU=follow-up.

Mean size, the sum of the longest diameter of liver metastases; numbers in parenthesis are percentages.

**Table 2:** Characteristics of liver operation

	**Group A, *N*=4**	**Group B, *N*=11**	***P*-value**
*Type of operation*			
Rt. Lobectomy	1	1	
Rt. lobectomy and WR	0	3	
Rt. lobectomy and RFA	0	1	
Lt. lobectomy and WR	1	0	
Segmentectoy and WR	0	2	
WR	1	2	
WR and RFA	0	2	
RFA	1	0	
Mean cycles of operation (range)	11 (8–16)	8 (4–12)	0.108

Abbreviations: RFA=radiofrequency ablation; Lt.=light; Rt.=right; WR=wedge resection.

Numbers in parenthesis are percentages.

**Table 3:** Cell death rate of each drug according to treatment response and liver resection in ATP-CRA-based chemotherapy

	**Treatment response**					**Liver resection**				
	**CR/PR**		**SD/PD**			**Yes**		**No**		
**Drug**	***N***	**CDR (%)**	***N***	**CDR (%)**	***P*-value**	***N***	**CDR (%)**	***N***	**CDR (%)**	***P*-value**
5-Fluorouracil	15	34.5	16	26.7	0.026	11	36.2	20	27.3	0.040
*Second drug*										
All	15	38.1	16	25.7	0.001	11	39.9	20	27.2	0.008
Oxaliplatin	8	41.0	12	24.5	0.013	7	41.6	13	25.5	0.033
Irinotecan	7	34.8	4	26.6	0.032	4	37.0	7	28.9	0.035

Abbreviations: ATP-CRA=adenosine triphosphate-chemotherapy response assay; CDR=cell death rate; CR=complete response; PD=progressive disease; PR=partial response; SD=stable disease.

**Table 4:** Univariate analyses of factors associated with treatment response and liver resection

	**Treatment response**			**Liver resection**		
	**CR/PR**	**SD/PD**		**Yes**	**No**	
**Characteristics**	***N*=22**	***N*=41**	***P*-value**	***N*=15**	***N*=48**	***P*-value**
Age (years)	57	56	0.684	55	57	0.307
						
*Sex*						
Male	12 (34.3)	23 (65.7)	0.906	6 (17.1)	29 (82.9)	0.156
Female	10 (35.7)	18 (64.3)		9 (32.1)	19 (67.9)	
						
*Primary tumour location*						
Rectum	8 (30.8)	18 (69.2)	0.562	6 (23.1)	20 (76.9)	0.909
Colon	14 (37.8)	23 (62.2)		9 (24.3)	28 (75.7)	
						
*Distant metastasis*						
Liver and lung	2 (28.6)	5 (71.4)	1.000	1 (14.3)	6 (85.7)	1.000
Liver only	20 (35.7)	36 (64.3)		14 (25.0)	42 (75.0)	
Number of liver lesions	7	9	0.078	8	9	0.658
Size of liver lesions (cm)	10.3	9.7	0.751	11.4	9.5	0.427
						
*Location of liver lesions*						
Bilobe	17 (32.7)	35 (67.3)	0.494	12 (23.1)	40 (76.9)	0.714
Unilobe	5 (45.5)	6 (54.5)		3 (27.3)	8 (72.7)	
						
Pretreatment CEA (ng ml^−1^)	74.8	73.0	0.939	81.1	71.3	0.710
						
*Chemotherapy regimen*						
FOLFOX	13 (28.3)	33 (71.7)	0.068	11 (23.9)	35 (76.1)	1.000
FOLFIRI	9 (52.9)	8 (47.1)		4 (23.5)	13 (76.5)	
						
*ATP-CRA*						
No	7 (21.9)	25 (78.1)	0.027	4 (12.5)	28 (76.2)	0.032
Yes	15 (48.4)	16 (51.6)		11 (35.5)	20 (64.5)	

Abbreviations: ATP-CRA=adenosine triphosphate-chemotherapy response assay; CEA=carcinoembryonic antigen; CR=complete response; PD=progressive disease; PR=partial response; SD=stable disease. Size=sum of the longest diameter of liver metastases.

**Table 5:** Multivariate analyses of factors associated with treatment response and liver resection

	**Treatment response (CR, PR)**			**Liver resection**		
**Characteristics**	**OR**	**95% CI**	***P*-value**	**OR**	**95% CI**	***P*-value**
Age (years)	1.1	0.9–1.2	0.105	1.1	0.9–1.3	0.080
						
*Sex*						
Male						
Female	1.0	0.2–4.8	0.963	1.8	0.4–9.0	0.434
						
*Primary tumour location*						
Rectum						
Colon	1.7	0.5–6.5	0.409	1.4	0.3–5.7	0.635
						
*Distant metastasis*						
Liver and lung						
Liver only	2.3	0.2–21.8	0.473	1.5	0.1–19.3	0.757
Number of liver lesions	1.4	1.1–1.8	0.010	1.2	0.9–1.4	0.056
Size of liver lesions (cm)	1.0	0.9–1.0	0.159	0.9	0.9–1.0	0.167
						
*Location of liver lesions*						
Bilobe						
Unilobe	1.4	0.3–7.4	0.691	1.2	0.2–7.9	0.812
						
Pretreatme CEA (ng ml^−1^)	1.0	0.9–1.0	0.605	0.9	0.9–1.0	0.376
						
*Chemotherapy regimen*						
FOLFOX						
FOLFIRI	3.3	0.8–14.3	0.113	1.3	0.2–6.8	0.751
						
*ATP-CRA*						
No						
Yes	15.2	2.4–96.3	0.004	16.8	2.0–139.6	0.009

Abbreviations: ATP-CRA=adenosine triphosphate-chemotherapy response assay; CEA=carcinoembryonic antigen; CI=confidence interval; CR=complete response; OR=odds ratio; PR=partial response; Size, the sum of the longest diameter of liver metastases.
